# Cross-Section Deformation and Bending Moment of a Steel Square Tubular Section

**DOI:** 10.3390/ma13225170

**Published:** 2020-11-16

**Authors:** Stanisław Kut, Feliks Stachowicz

**Affiliations:** Department of Materials Forming and Processing, Rzeszow University of Technology, al. Powst. Warszawy 8, 35-959 Rzeszów, Poland; stafel@prz.edu.pl

**Keywords:** shaping, bending, box profile, constructional S235JR steel, deformation, FEM

## Abstract

When bending thin-walled profiles, significant distortion of the cross-section occurs, which has a significant impact on the course of the bending moment characteristics and on the value of allowable bending curvatures. This paper presents the results of experimental and numerical modeling of the box profile bending process, which was carried out in order to determine the dependence of the cross-sectional shape and bending moment of bending curvature. Extensive numerical calculations were used to model the process of shaping a square pipe from a circular tube and to model the bending process, especially when taking into account the effects of such a deformation path. The pure bending moment characteristics and the deformation of the cross-section were performed for a 25 × 25 × 2 mm square tube made of S235JR structural steel. The innovative approach for determining the parameters of cold bending square tubes pertained to considering the stress state in the preserved material in individual areas of their cross-section. The results of numerical modeling—after considering the history of deformation (i.e., the process of forming a square pipe from a pipe with a circular cross-section)—gave a satisfactory agreement with the results of experimental tests, both in terms of the degree of pipe wall deflection and the characteristics of the bending moment.

## 1. Introduction

Rectangular steel tubes are widely used as structural elements in numerous applications. The advantages of rectangular pipes over circular tubing include the fact that they are easy-to-fit regarding the direction, produce a high torque when twisting, and reduce the weight of structured parts [1]. Rectangular pipes are usually produced from a welded round tube using a cold drawing trough and a head, which is comprised of idle rolls [2,3]. However, many experimental works and theoretical studies, including numerical calculations [4,5], are devoted to the phenomena that occur during the formation of rectangular pipes, but many issues still need to be worked out. For example, differences in mechanical properties result from the formation process observed around the periphery of rectangular pipes [6], especially in their corners, which may probably affect the metal flow process in the production of bent products.

**Figure 1:**
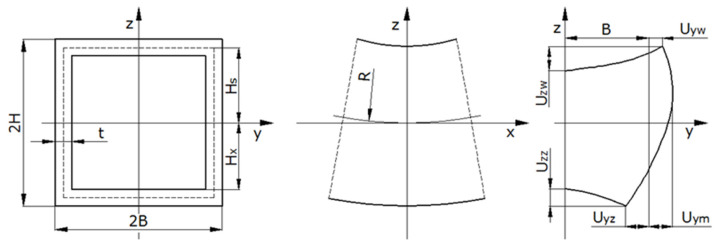
Characteristic tube dimensions and displacements of the deformed cross section [8].

During the elastoplastic bending process of thin-walled profiles, their cross-sections are subjected to distortions, which strongly affect the bending moment characteristics, the value of the allowable (minimum) bending radius and the amount of the springback e.g., [7,8,9,10]. In the case of bending pipes with a circular cross-section, the parameters that describe the deformation degree of the cross-section is described by an indicator called ovalization. In the case of bending pipes with a rectangular cross-section (box profiles), it is not possible to apply a similar unique parameter due to the complex shape of the cross-section after bending, which is significantly different from the initial cross-section. The cross-sectional deformation that occurs during bending leads to the pipe collapsing after exceeding its critical curvature value [7]. The observed changes in the geometry of the cross-section of rectangular tubes and the determination of the impact of these changes on the value of other parameters characterizing the bending process have been the subject of many studies. Based on the numerical modelling results of fine element method (FEM), Chen and Masuda [9] found that the flattening ratio can be expressed by a sole function of nondimensional curvature, independent of tube thickness in elastic bending. During plastic bending, flattening is influenced by tube thickness and the material parameters of yield stress and strain hardening; however, these effects are very small. The issues related to the bending process of box profiles are presented in numerous publications, including experimental studies [8,11], theoretical considerations based on the deformation theory of plasticity [12,13,14,15], numerical modelling with the use of FEM [16,17,18,19], and the use of artificial intelligence identification [20]. Using the deformation theory of plasticity and the energy method that Poulsen et al. [14] built, the suck-in of the external flange of the profile was almost independent of strain hardening and the material’s yield stress. Observations and measurements of the bent cross-section shape of the box-section enabled us to establish relationships that determine the dependence of the cross-sectional when forming the bending curvature [8]. The following indices are assumed for the description of the shape of a cross-section after bending (Figure 1):

U_zw_: deflection of the inner horizontal wall,

U_zz_: deflection of the outer horizontal wall,

U_yw_: deflection of the inner profile corners,

U_yz_: deflection of the outer profile corners,

U_ym_: maximum lateral displacement of points of the wall beyond the original cross-section.

The horizontal walls were those that normalized to the bending plane; the vertical walls were those parallel to the bending plane. ‘Inner’ and ‘outer’ refer to the direction forward and away from the bending axis, respectively.

The purpose of this work was to verify the correctness of the numerical model used to determine the changes in the geometry of the box profiles’ cross-section, as well as the relationship between the bending moment and the bending curvature based on the results of experimental studies. An additional purpose of the research was to determine the effect of technological deformations and internal stresses created in the plastic shaping of a round tube into a square section on selected bending parameters of a 25 × 25 × 2 mm square steel pipe. The scope of the research work included:

1. FEM numerical modelling during the cold plastic shaping process of a round tube with an external diameter (d = 30 mm) and a wall thickness (g = 2 mm) into a square cross-section pipe.
2. FEM numerical modelling of the process of forming a square pipe with a pure bending moment. Model A took into account the technological deformations and residual stresses created during shaping of a square pipe. Model B did not take into account the impact of technological deformations and internal stresses created during shaping of a square pipe.
3. Experimental four point bending of a 25 × 25 × 2 mm square pipe was tested to verify the results of numerical calculations.

## 2. Material and Experimental Procedure

The study was carried out on a 25 × 25 × 2 mm box profile, which was cold shaped from a welded round tube made of constructional S235JR steel. The samples prepared from the profile wall were tested under uniaxial tension using the Hollomon strain–stress relationship in the form of $\sigma =530\varepsilon^{0.044}$, which showed significant reproducibility. Other tensile parameters were as follows: yield stress R_e_ = 388 MPa; ultimate strength R_m_ = 486 MPa; Young modulus E = 199 GPa; Poisson’s ratio ν = 0.3.

**Figure 2:**
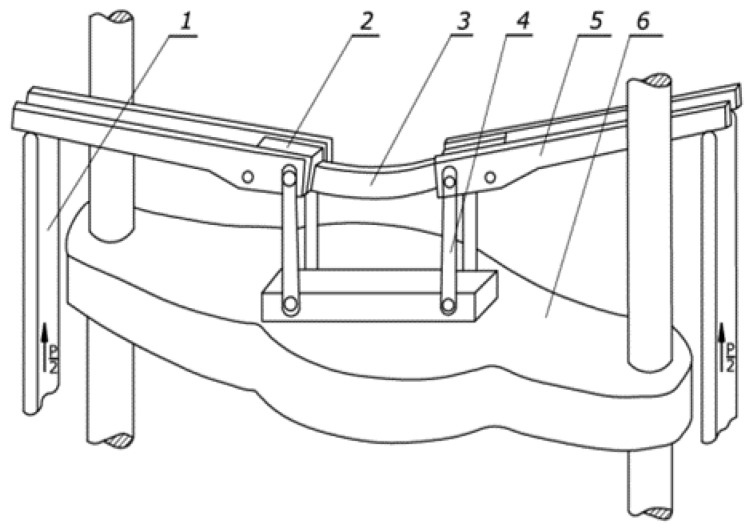
Laboratory four point bending test device [8]: 1—lever, 2—clamping jaws, 3—sample, 4—connector, 5—bending arm, and 6—traverse of the testing machine.

The experimental tests were carried out with the help of a device mounted on a testing machine (Figure 2). It should be noted that the sample was placed in the holders in such a way that the wall with the weld was a vertical wall, indicating that the joint material was in the neutral layer. The bending on four supports ensured that the pipes were loaded with a pure bending moment, without the use of lateral forces. During bending, the deflection of horizontal (inner and outer) and vertical profile walls, as well as the bending force, were measured continuously. Then, the bending moment value was calculated. For several selected bending steps, measurements were made of the tube curvature and the displacement of characteristic points of individual walls of the cross-section, i.e., the value of the section widening factor 2Uym, the value of the deflection of the internal horizontal wall Uzw, and the external horizontal wall Uzz.

## 3. Numerical Calculations

**Figure 3:**
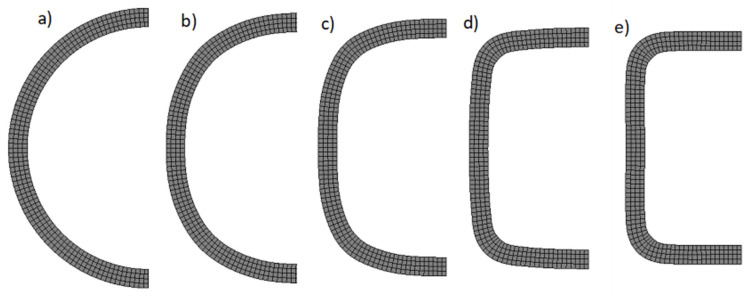
Change in the shape of the cross section of the pipe in the subsequent stages of profiling: (**a**) round pipe, (**b**–**d**) intermediate shapes; (**e**) square pipe.

In order to determine the distributions and values of plastic deformations and residual stresses prevailing in the pipe after the cold shaping process, we constructed a numerical model of the cold profiling process. The starting material was a round pipe with an external diameter (d = 30 mm) and a wall thickness of 2 mm, made of S235JR constructional steel, which was profiled to obtain a 25 × 25 × 2 mm square pipe. Due to the fact that the pipe length did not change during profiling, the profiling analysis was carried out in a flat deformation state. Due to the occurrence of plane symmetry, the numerical model was made for half of the cross-section. The shaping tools were modelled as perfectly rigid. Quad 4 elements type 11 were used to discretize the deformable body [21]. In order to improve the behavior of these elements during bending, an additional interpolation function was applied during the assumed strain procedure [21]. An elastic–plastic model of the pipe material with non-linear strengthening described by the Hollomon power equation was adopted. Isotropic properties of the pipe material were assumed for both elastic and plastic deformations. The strain curve was introduced into the program in a tabular form. The entered coordinates of the strain hardening curve were calculated on the basis of the pipe material parameters, which were determined on the basis of the uniaxial tensile test: Re = 388 MPa, Rm = 486 MPa, C = 530 MPa, and n = 0.044. To describe the friction between the tools and the profiled pipe, we used the Coulomb friction model with a friction coefficient of 0.1. Numerical modelling was performed using a commercial MSC.MARC/Mentat system, which is considered one of the most advanced programs for FEM analysis, and particularly for modelling nonlinear and contact issues. The change in the shape of the cross-section during pipe shaping is shown in Figure 3. The input material is a pipe with a circular cross-section (Figure 3a), which in the technological process is constructed by longitudinal rolling. Such a pipe is also formed by longitudinal rolling and, in subsequent stages, it takes intermediate shapes (Figure 3b–d) until the required square section is obtained (Figure 3e).

**Figure 4:**
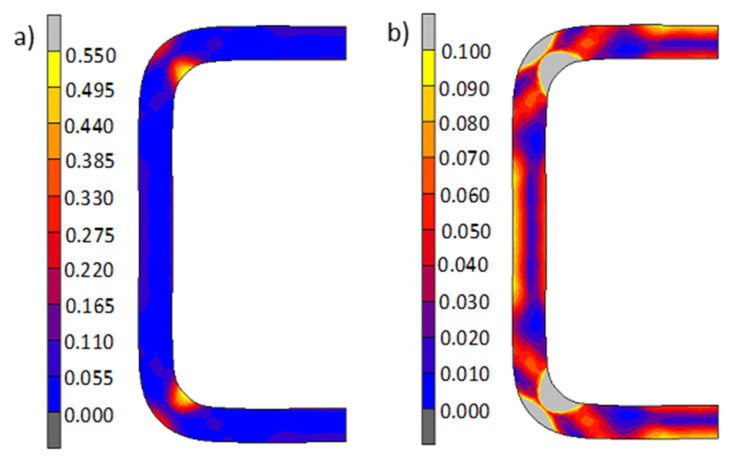
Distribution and values of the total equivalent plastic deformations in the pipe material after cold shaping; value scale: (**a**) strain range (0–0.55) and (**b**) strain range (0–0.10).

As is commonly known, the properties of elements formed via cold working are significantly affected by the strain hardening phenomenon, which is when a material’s formability is reduced. Figure 4 shows the distribution and values of the total equivalent plastic strain in the pipe material after shaping. The highest values of plastic strain occur in the corners, especially in the area of the internal radii, where their calculated values reach 0.55 (Figure 4a). Quite significant material hardening points are also deformations in other areas of the material. The inequality of the distribution of plastic strains on the cross-section of the pipe is even more evident when deformations are smaller than 0.1, as shown in Figure 4b.

**Figure 5:**
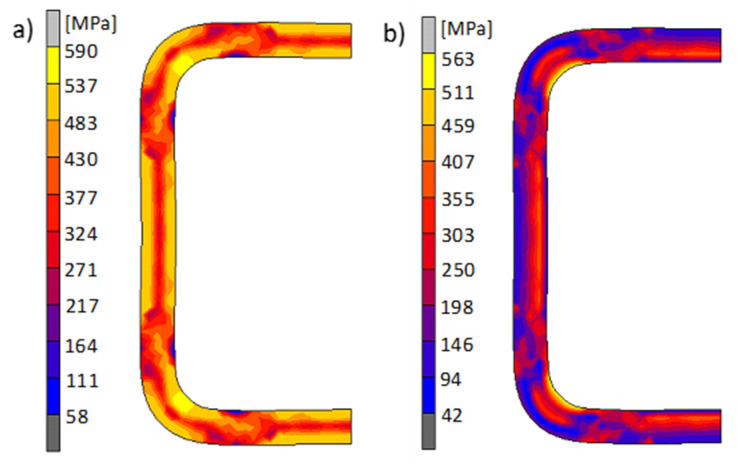
Distribution and values of the Huber–Mises equivalent stresses in the material of the shaped pipe: (**a**) in the final shaping phase and (**b**) after the shaping process (internal stress).

The second important parameter that has a significant impact on the properties of components shaped via cold forming are residual stresses that prevail in the material after the technological process [16]. Residual stresses are the result of heterogeneous plastic deformations in the material after its formation. The greater the heterogeneity and gradient of plastic deformations, the higher the material stress values, which may be close to the yield point in accordance with the course of the strain curve. Figure 5a shows the distribution and values of Huber–Mises equivalent stresses in the final shaping phase are shown in Figure 5a, while Figure 5b shows the distribution of these stresses that prevail in the material after the technological process. As can be seen, the largest values of equivalent stresses occur in the areas of the largest deformation gradients and amount to about 590 MPa in the final shaping phase. In turn, the residual stresses in this area reach the highest value of about 563 MPa, i.e., only slightly lower (about 27 MPa) than the yield point. Both plastic deformation and own stress have a significant impact on the material after the technological process. As demonstrated after the cold pipe profiling process, their share is significant. For this reason, we carried out an analysis of the impact of these parameters for a square pipe bending process.

**Figure 6:**
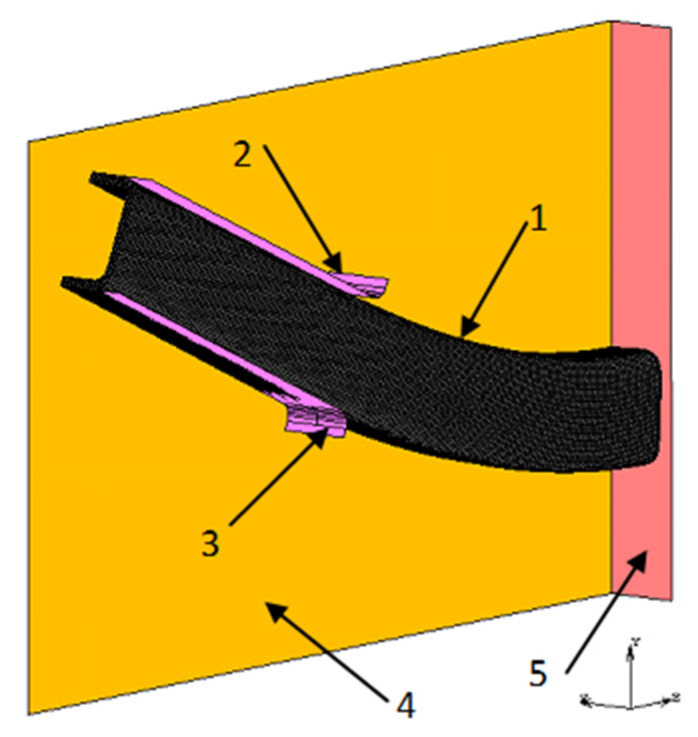
3D model of the analyzed bending process: 1, bent beam with a fine element (FE) mesh; 2 and 3, surfaces of the clamping jaw; 4 and 5, longitudinal and transverse symmetry planes.

Figure 3e shows the FEM analysis of the 25 × 25 × 2 beam bending process, which was carried out in two variants. In the first variant (Model A), both residual stresses and plastic deformation that prevail in the beam material after the technological process were taken into account (Figure 4 and Figure 5b), whereas in the second variant (Model B), only the previously calculated cross-sectional shape was taken into account without considering residual stresses and deformations. Other parameters of the spatial stress state used in both models were the same. 3D models of the beam bending were made on the basis of bending device geometry, which was used in the experimental research described later in the article. Due to the occurrence of two plane symmetries while bending the tested beam along the beam axis and perpendicular to the cross-section in the middle of the distance between the supports, the numerical models were simplified to ¼ of the experimental model. The beam was defined as a deformable body, while the elements of the device in contact with it were perfectly rigid. To discretize the deformable body, 8-node hex-shaped elements of type 7 were used [21]. The size of the elements on the cross-section of the beam was 0.5 × 0.5 mm, while on its length it was 1 mm, which gave a total of 68,400 finite elements. The 3D model during bending, together with the mesh and defined bodies in contact, as well as planes of symmetry, is shown in Figure 6. An elastic–plastic model for beam material with a non-linear strain hardening curve described by the Hollomon power equation was adopted. The material parameters were the same and defined in the same way as during the simulation of the square pipe shaping process. In all models, the evolution of the plastic surface came as a result of the strain hardening phenomenon, which was described using the isotropic hardening model. The calculations used the Huber–Mises–Hencky plasticity condition, the associated Prandtl–Russ plastic flow law, and the implicit integration of differential equations over time with the Newton–Raphson method.

**Figure 7:**
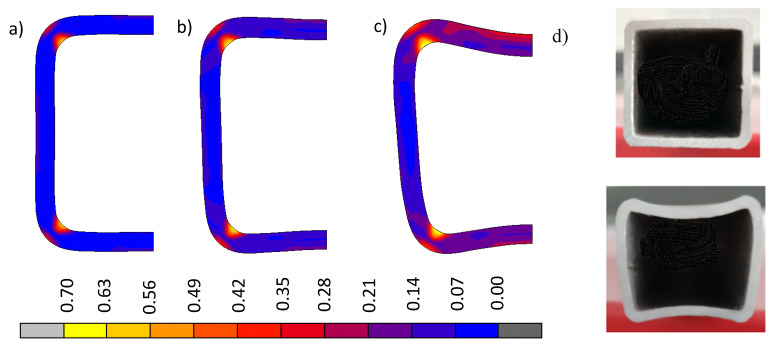
The change in the cross-sectional shape of a bent beam (model A) and the distribution of total substitute plastic deformations for: (**a**) 1/R = 0 m^−1^, (**b**) 1/R = 4 m^−1^, (**c**) 1/R = 6 m^−1^, and (**d**) cross-section templets before (upper) and after bending the local break (lower).

**Figure 8:**
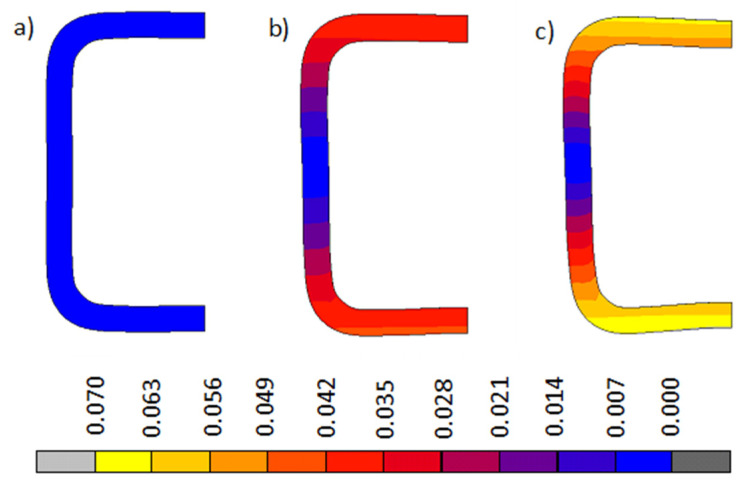
Change in cross-sectional shape of the bent beam (model B) and distribution of total substitute plastic deformations for: (**a**) 1/R = 0 m^−1^, (**b**) 1/R = 4 m^−1^, and (**c**) 1/R = 6 m^−1^.

Figure 7 and Figure 8 show a comparison of the changing cross-sectional shape of the bent beam and the distribution and values of plastic deformations for Model A and Model B respectively. Taking into account technological deformations and residual stresses created when cold shaping a square pipe (model A), results had a much greater degree of deformation in the pipe’s cross-section than when we did not take into account the impact of technological deformations and internal stresses created during the shaping (model B). Photographs of the pipe cross-sections before and after the bending process (Figure 7d) confirmed the above mentioned statement. The shape and size of the deflection of the cross-section walls observed in the experimental sample photograph were similar to the image of the cross-section obtained via numerical modeling, which considered technological deformations and residual stresses created when cold shaping a square pipe (model A).

**Figure 9:**
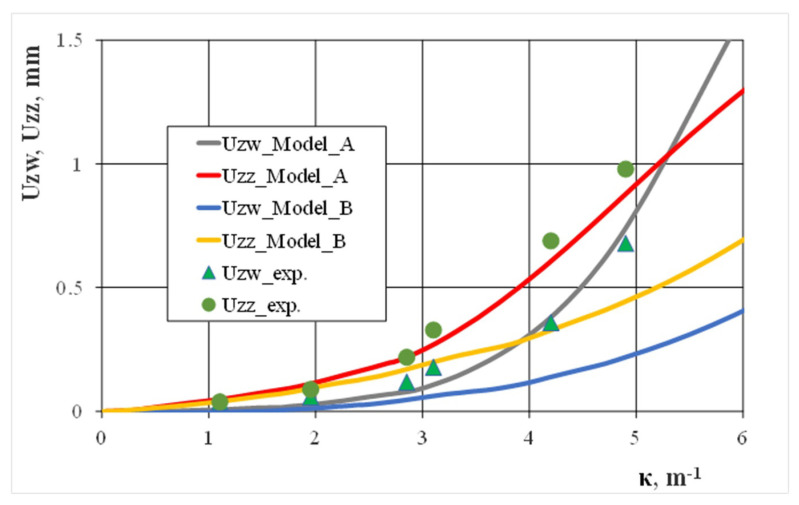
The effect of bending curvature when deflecting the horizontal walls of the 25 × 25 × 2 square steel pipe.

**Figure 10:**
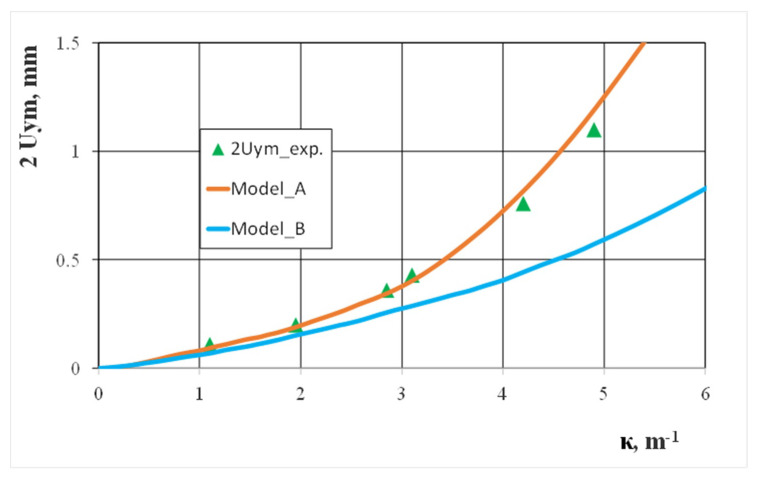
The effect of bending curvature when deflecting the vertical walls of the 25 × 25 × 2 square steel pipe.

With the measurements results carried out during the experimental studies, we compared numerical calculations for the displacement values of selected points in the profile cross-section, which occurred during a curvature increase. If the horizontal walls deflection degree internally and externally changed, good compliance of the calculation and experimental results were obtained (Figure 9). However, this was only when we considered technological deformations and residual stresses created when cold shaping a square pipe (model A). Similarly, large differences between the numerical calculation results and experimental results were found when we analyzed the degree of the vertical wall bending (Figure 10). This was because it did not take into account the technological deformations and stresses created when cold shaping a square pipe (model B). During experimental bending tests, we observed that bending the pipe above the curvature κ > 6.0 caused a localization of the deformation on the internal horizontal wall and led to the pipe’s local collapse. The above observation was confirmed by the numerical calculation results carried out according to model A, which indicated an intensive increase in the deflection of the internal horizontal wall for large bending curves. The location of the deformation in the pipe cross-section resulted in its buckling and caused a significant share of compressive stresses. It should be noted that deflecting the vertical walls in the profile cross-section slightly affected the moment of inertia value, which is what determined the bending moment value.

**Figure 11:**
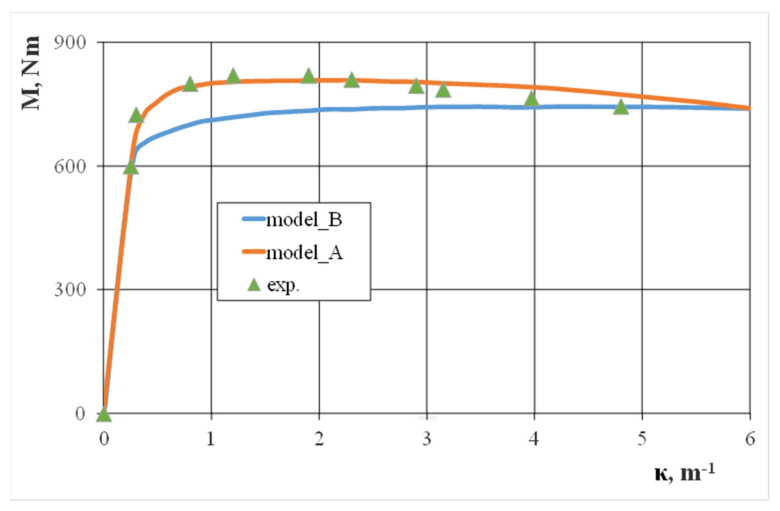
The effect of bending curvature on the bending moment of the 25 × 25 × 2 square steel pipe.

As expected, the improved modelling of the numerical degree of distortion for the bent square tube’s cross-section had good compliance with the bending curve, which was experimentally determined via numerical calculations (Figure 11). This occurred during technological deformations and residual stresses after making a square tube from a circular tube via the cold forming process (model A). The bending characteristics that were determined numerically in accordance with the assumptions for model B differed from the bending characteristics determined experimentally, especially in the small bending curvature area. Only in the bending curvature range of κ > 3.0 m^−1^ were the bending moment values numerically calculated. Over the wide range of bending curvatures, the bending moment values did not change much, which was partially a result of the pipe material’s slight strain hardening. On the other hand, even a slight strain hardening should increase the value of the bending moment. At the same time, the decrease the cross-section’s moment of inertia value was a result of the walls deflecting, especially the horizontal ones, which completely eliminated the deformation strengthening effect. For bending curvatures κ > 6.0 m^−1^, the pipe collapsed locally, which was accompanied by a sharp drop in the bending moment value.

## 4. Conclusions

Our results made it possible to describe changes in the shape of a square tube’s cross-section as a function of bending curvature. Further, our research determined the impact of these changes on force characteristics, i.e., the value of the bending moment on bending curvature. As a result of cross-section distortion for a curvature of κ > 2.0 m^−1^ in the initial bending phase, we observed a decrease in the bending moment value. For a curvature of κ > 6.0 m^−1^, the pipe collapsed locally. Satisfactory agreement of the experimental results and numerical calculations was obtained by considering the technological deformations and residual stresses created during the cold plastic shaping process of a round tube into a square cross-section pipe.
